# Conformational Selection and Induced Fit Mechanisms in the Binding of an Anticancer Drug to the c-Src Kinase

**DOI:** 10.1038/srep24439

**Published:** 2016-04-18

**Authors:** Maria Agnese Morando, Giorgio Saladino, Nicola D’Amelio, Encarna Pucheta-Martinez, Silvia Lovera, Moreno Lelli, Blanca López-Méndez, Marco Marenchino, Ramón Campos-Olivas, Francesco Luigi Gervasio

**Affiliations:** 1 Structural Biology and Biocomputing Programme, Spanish National Cancer Research Centre (CNIO), c/Melchor Fernandez Almagro 3, 28029, Madrid, Spain; 2 Institute of Structural and Molecular Biology, University College London, London WC1E 6BT, United Kingdom; 3 Department of Chemistry, University College London, London WC1E 6BT, United Kingdom; 4 Chemistry Department, University of Florence, 50019, Sesto Fiorentino (FI), Italy; 5 Spectroscopy and NMR Unit, Spanish National Cancer Research Centre (CNIO), c/Melchor Fernandez Almagro 3, 28029, Madrid, Spain

## Abstract

Understanding the conformational changes associated with the binding of small ligands to their biological targets is a fascinating and meaningful question in chemistry, biology and drug discovery. One of the most studied and important is the so-called “DFG-flip” of tyrosine kinases. The conserved three amino-acid DFG motif undergoes an “in to out” movement resulting in a particular inactive conformation to which “type II” kinase inhibitors, such as the anti-cancer drug Imatinib, bind. Despite many studies, the details of this prototypical conformational change are still debated. Here we combine various NMR experiments and surface plasmon resonance with enhanced sampling molecular dynamics simulations to shed light into the conformational dynamics associated with the binding of Imatinib to the proto-oncogene c-Src. We find that both conformational selection and induced fit play a role in the binding mechanism, reconciling opposing views held in the literature. Moreover, an external binding pose and local unfolding (cracking) of the aG helix are observed.

Molecular recognition plays a fundamental role in all biological processes. Understanding it in atomic detail is of great importance as it could lead to more effective and less toxic drugs. Most of the current knowledge stems from high-resolution crystal structures, time resolved spectroscopy and atomistic molecular dynamics simulations, which made clear that molecular recognition is a very dynamical event. Both the ligand and the target can change shape to achieve a tight fit. The two limiting mechanisms describing this dynamical adaptation, the “induced fit”^1^ hypothesis and the “conformational selection” hypothesis^2^,^3^ have been now observed in different systems and it is increasingly clear that both play a role^4^,^5^.

An interesting case, which has recently attracted much attention, is that of the powerful anti-leukemic drug Imatinib (also know as Gleevec), that revolutionized cancer treatment since its discovery in 2001^6^. The drug has a 2300-fold lower inhibitory power towards the c-Src tyrosine kinase (TK)^7^ relative to its specific target, the c-Abl tyrosine kinase^8^,^9^, despite the high sequence identity (47%). Both conformational selection and induced fit effects have been invoked to justify such a dramatic difference. Imatinib binds to an inactive conformation, where the aspartate (Asp_404_ in c-Src) of the conserved Asp-Phe-Gly motif (DFG), located at the N-terminal end of the long activation loop (A-loop), points outwards from the ATP cavity^10^ (Fig. 1). Thus, at first, the selectivity of Imatinib towards c-Abl has been attributed to the inability of c-Src of assuming this peculiar “DFG-out” conformation^11^,^12^. However, subsequent X-ray structures of Imatinib co-crystallized with c-Src revealed a DFG-out conformation and an extremely similar binding mode^13^. A DFG-out conformation has since been observed in many PKs^14^ and a functional role for the in-to-out flip has been proposed^10^.

The different selectivity was then proposed to be due to the thermodynamic penalty of the DFG-out state in c-Src^10^,^13^,^15^, which prompted an extensive study of this conformational change by computer modeling and simulations^16^,^17^,^18^,^19^,^20^,^21^. Using state-of-the-art free energy methods, we have proposed that the most important contribution to the binding free energy difference of Imatinib to c-Src and c-Abl is, indeed, the stability of the DFG-out conformation^15^, according to a “conformational selection” mechanism. The lower stability of the DFG-out conformation in c-Src was also confirmed subsequently by a different computational approach^21^. However, an alternative “induced fit” mechanism has been recently proposed based on the interpretation of NMR spectra and stop-flow fluorescence^22^. The data were interpreted to be consistent with a two step process. The authors proposed that the two steps are a fast binding of the drug, followed by a slow conformational change. Only the final state, after the slow “induced fit” is able to bind Imatinib in the nanomolar range (the initial bound state has K_*d*_ in the *μM* range) and the difference in population of this second better-binding state is estimated to determine, on its own, a 1000-fold difference in affinity. The nature of the proposed “induced fit” step, however, is still debated, also due to an updated model from the same experimental group^23^, that, more in line with earlier theories^24^, stresses the importance of the conformation of the P-loop (also known as g-loop or glycine-rich loop, Fig. 1). In the case of Abl and its drug-resistant mutants, we recently proposed a binding mechanism in which two different conformational changes play a role, providing a consistent interpretation to the recent experimental and computational findings^25^.

Here, to fully characterize the binding mechanism of Imatinib to c-Src, we combine experimental and computational techniques, including *μ*s long unbiased simulations, free energy calculations, high resolution NMR experiments and surface plasmon resonance. In so doing, we fully sample the conformational changes associated with the binding and shed some light on the relative roles of “conformational selection” and “induced fit” effects.

Our combined spectroscopic and computational results suggest a complex association mechanism. On the free energy surface two alternative binding paths coexist in which at room temperature conformational selection plays a major role and induced fit a minor one. Conformational changes and meta-stable binding modes contribute to an heterogeneous ensemble of intermediate states. Distant structural elements such as the A-loop, the *α*C, *α*G and *α*D helices are involved in coordinated movements thanks to a network of electrostatic interactions involving the conserved residues Glu_310_, Arg_409_, Asp_404_ and Lys_295_ What is more, both simulation and experiments agree on a significant role of local unfolding, supporting predictions based on energy landscape theory^26^,^27^.

## Results

**Figure 1:**
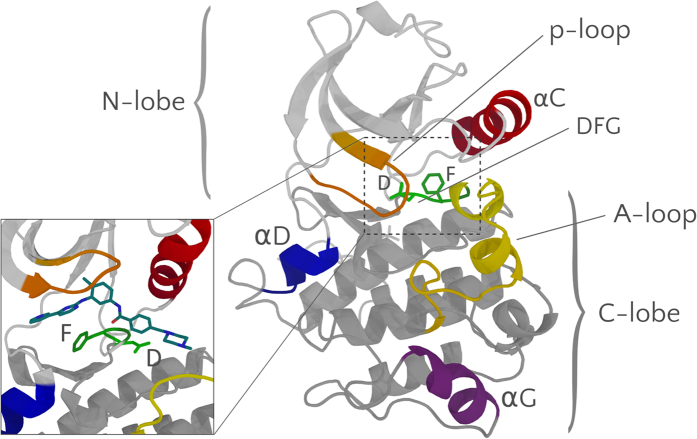
Structure of the kinase domain of c-Src with a detailed view of the DFG motif in the DFG-in apo conformation (DFG represented as green sticks; PDB ID: 2SRC). The inset shows the DFG-out Imatinib-bound form (the DFG and Imatinib are shown as green and blue sticks, respectively; PDB ID: 2OIQ).

**Figure 2:**
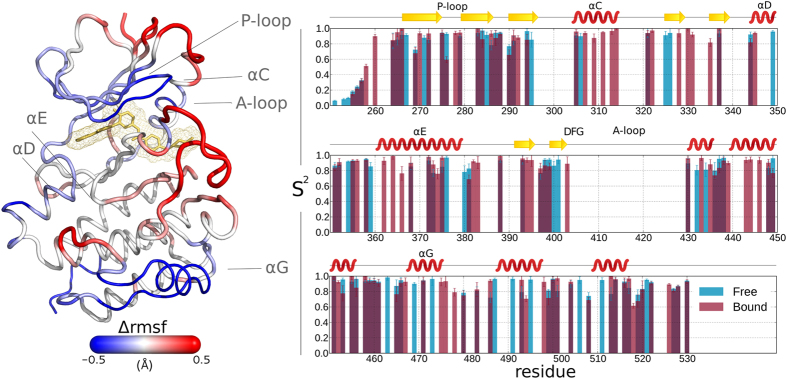
c-Src Dynamics, (left) difference in the *Cα* RMSF (Δ_*rmsf*_) between the apo and ligand-bound forms of c-Src averaged over 1 *μ*s-long MD simulations. Low (blue) values correspond to regions that are more rigid (lower RMSF) in the bound form, high (red) values correspond to regions that are more flexible in the bound form. (right) Experimentally determined order parameters *S*^2^ for the apo (blue) and bound (red) forms.

The 2D ^1^H, ^15^N-TROSY NMR spectrum of the ligand-free (apo) form of c-Src is shown in SI Fig. S1. Out of 268 backbone amides, 196 signals are visible, of which 179 (91%) were assigned (BMRB entry 25756). Most of the signals corresponding to the A-loop (Figs 1 and 2), the *α*C helix and some of those corresponding to the *α*G helix are missing.

The absence of signal might be due either to a fast exchange with the solvent or to a slow conformational equilibrium in the ms-*μ*s time-scale leading to signal broadening beyond detection. As the solvent exchange rate is highly dependent on pH, its extent can be inferred by acquiring the spectrum at different pH^28^. Indeed, increasing the pH from 6.4 to 8 causes the disappearance of many signals corresponding to solvent-exposed amide protons, as expected from a fast exchange of amino protons with the solvent (SI Fig. S2). On the contrary, decreasing the pH to 5 has little effect on the number of visible signals, while significantly reducing the stability of the protein. The reduced stability might be in part due to the slightly higher mobility of the DFG motif in the protonated Asp state^15^, as also observed in the case of Abl^10^ and FGFR1^14^. Thus, a slow conformational equilibrium is expected to be the main reason for the absence of many signals in the spectrum recorded at pH 6.4. A similar effect was previously reported for the A-loop of the p38 kinase^29^.

In the TROSY spectrum of the Imatinib-bound c-Src many of the missing signals in the ligand-free form become detectable^30^, in agreement with a substantial alteration of the conformational dynamics upon ligand binding (SI Fig. S2).

### Fast dynamics of the apo and Imatinib-bound forms

To investigate the effect of Imatinib binding on the fast dynamics of c-Src, we performed long (1 *μs*) MD simulations and NMR relaxation measurements on the free and Imatinib-bound forms of c-Src. In Fig. 2 and SI Fig. S4 we report the C_*α*_ Root Mean Square Fluctuation (RMSF) and the experimentally determined *S*^2^ order parameters corresponding to the two forms. While the two parameters cover mostly overlapping time-scales, the RMSF, performed on the 1 *μ*s-long trajectories is sensitive to changes happening in the ps to hundreds of ns time-scale, while *S*^2^ is limited to motions faster than the overall tumbling time. A direct comparison of the computed and experimental S^2^ parameters is reported in SI Fig. S5 and shows a good agreement.

In the apo form the largest RMSF is observed for the region of the *α*G helix, whose orientation fluctuates on a time-scale of hundreds of ns. In the bound form lower RMSF values (i.e. a rigidification) are observed for regions directly interacting with the drug, as the N-lobe *β*-sheets and loops, and in particular for the glycine-rich P-loop. The hinge region dividing the two lobes, where Imatinib interacts with residues Thr_338_ and Met_341_, is also rigidified, as the central region of the *α*C helix, near the drug. Surprisingly, the region encompassing the *α*G helix and the nearby loops, albeit not in direct contact with the inhibitor, experiences the most significant rigidification. On the contrary, the A-loop and the initial segment of the *α*C helix are more flexible in the bound form. The average conformation of the A-loop in the bound form is similar to that found in the crystal structure of Imatinib-bound c-Abl. Throughout both simulations the salt bridge between Glu_310_ and Arg_409_, typical for the so-called *α*C-out conformation^13^, is engaged.

To characterize the motions faster than the overall tumbling, the R_1_ and R_2_^15^*N* NMR relaxation rates and the ^1^*H*, ^15^*N* heteronuclear NOE of backbone amide nitrogen were measured at 700 MHz. The software TENSOR2^31^, was used to analyze the data, yielding the isotropic rotational correlation time *τ*_*c*_ and the Lipari-Szabo sub-nanosecond order parameters (S^2^)^32^ (see SI).

Both structures appear to be relatively rigid in the sub-*μ*s time scale with an average S^2^ of about 0.85 in the apo form and 0.87 in the Imatinib-bound form (SI Fig. S3). The only exception is the unstructured N terminus and the two loops following the *α*G-helix that are more flexible (see Fig. 2, again in good agreement with simulation data in SI Fig. S5). The general agreement between observed and calculated fast motions can be also seen in the comparison of NMR- and simulation-derived S^2^ parameters. The average deviation is less than 9% (SI Fig. S5). The most significant discrepancy is found in the region of the *α*G helix in c-Src apo form. The NMR data indicates lower fluctuations for the visible signals assigned to this region. This is due to the slower character of these re-orientation motions that in the simulation happen in the hundreds of ns to *μ*s regime, well beyond the NMR-determined tumbling time of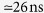.

Overall, our NMR results do not show large changes in the fast dynamics upon ligand binding. Simulations indicate that the rigidification of some regions is compensated by a larger flexibility in other areas. Thus the entropy associated to fast motions should not play a major role in the binding mechanism.

Instead, we expect that many structural elements, including the *α*G and *α*C helix and the A-loop undergo large conformational changes in the *μ*s-ms time scale. This is strongly suggested by the values of the R_1_R_2_ product^33^, the exchange contribution to relaxation R_*ex*_ parameter^32^, the borderline deviations from random coil values of the chemical shift index^34^ (CSI, SI Fig. S3), the many missing signals in the spectrum and the slow conformational changes observed in the MD simulations.

### Changes in the chemical shifts

**Figure 3:**
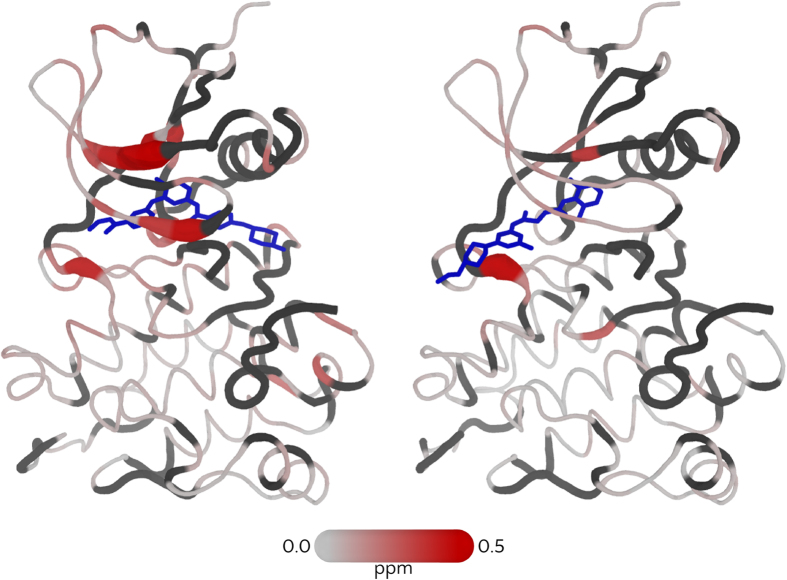
Difference of combined ^1^H, ^15^N chemical shift Δ*δ* for Src upon binding of Imatinib (left) and Dasatinib (right). Ligands are shown as blue sticks. Residues for which data is not available are shown in dark grey.

The changes in chemical shifts due to the binding of Imatinib are reported in Fig. 3 and SI Fig. S6. The residues that become visible only in the bound-form are shown in Fig. S6 and include many residues of the *α*C and *α*G helices as well as the regions closest to Imatinib of *α*E, *α*F and *α*I. The appearance of signals from the *α*C helix and from two flanking beta strands, indicating a significant change in their conformational dynamics, is of particular interest. This helix is known to reposition in the bound form (*α*C-in) through the formation of a salt bridge between Glu_310_ and Lys_295_^35^. The *α*C helix appears to be essential in modulating the function of the protein and its movement is connected with the conformational changes of the activation loop^36^.

Most of the signals corresponding to the A-loop remain undetectable. A noticeable exception is Asp_404_, the first residue of the DFG motif, whose signal appears in the bound spectrum. As Imatinib stabilizes the DFG-out conformation, the appearance of the Asp signal is presumably due to the suppression of the in-out equilibrium as in the case of the p38 kinase^29^. The absence of the signals corresponding to the Phe_405_ and Gly_406_ of the DFG in the bound form is probably due to some degree of residual motion connected to the A-loop dynamics combined with the perturbation of the chemical shift that could be induced by the large magnetic anisotropy of Phe_405_. This hypothesis is supported by the larger B-factors registered for Phe_405_ and Gly_406_, compared to Asp_404_, in the X-ray structure of the bound form^13^.

The appearance of the signal corresponding to Leu_472_ upon binding is in agreement with MD simulations, showing a rigidification of the *α*G helix. The large magnetic anisotropy of the aromatic rings of Imatinib leads to sizeable chemical shift changes. Their sign allows to locate the compound in the crystallographic pose (SI Fig. S6). Some of the signals appearing in the bound form correspond to the secondary pocket proposed on the basis of the free energy calculations (see below). This indicates rigidification of the area due either to a direct, albeit transient, interaction with the drug or to an indirect allosteric effect. A dynamical connection of the two sites would be in agreement with an at least partial induced fit effect, as discussed below.

Chemical shift data were compared with those obtained using a DFG-in binding inhibitor, namely the second generation tyrosine kinase inhibitor Dasatinib^37^. Figure 3 shows the perturbations induced by Dasatinib on the amide chemical shifts of c-Src, in the same experimental conditions. Also in this case, the most affected region is the binding pocket. The main difference between the two is that Imatinib induces a much larger effect on the nearby beta strands of the N-terminus. This can be easily understood by looking at the crystal structure: in the case of Imatinib, the four aromatic moieties (which induce large changes in their surroundings) are placed underneath the beta sheet of the N terminus and oriented towards it (see Fig. 3). The signs of the deviations (which mostly indicate shielding, see Fig. S6) also support this hypothesis. On the contrary, Dasatibib induces effects only on the beta strand 3, to which one of its aromatic rings is close. Also the rigidification of mobile parts of the protein seems to be less pronounced in the case of Dasatinib (Fig. S6). A few signals reappear in the presence of the drug, but not to the extent found in Imatinib. This is also not too surprising, as Imatibib interacts with a large part of the *α*C helix and contributes to its stabilization. This is not the case with Dasatinib, which points in the opposite direction.

### Free energy profile and binding mechanism

**Figure 4:**
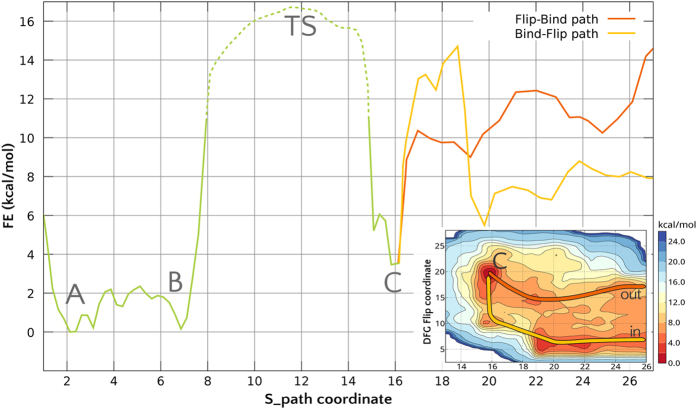
Imatinib binding free energy profile along the *S*_*path*_ variable, which defines an optimal association coordinate. The most relevant minima and the transition state are labelled and discussed in the text. The area of the transition state is represented as a dashed line due to a larger uncertainty. The two profiles in the region 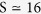 to 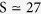 (secondary pocket to the unbound state) correspond to the conformational selection mechanism (red line, “flip-bind”) and to an induced fit mechanism (yellow line, “bind-flip”). The 2D FES in the inset shows the dependence of the free energy from the DFG-flip coordinate.

The free energy profile associated to the (un)-binding of Imatinib from c-Src obtained by extensive Parallel Tempering Metadynamics (PT-MetaD)^38^ simulations is shown in Fig. 4.

The profile is characterized by a deep and broad energy basin in which the crystallographic bound state corresponds to the deepest minimum (A in Fig. 4). An almost equally stable state (B) is found within the same basin. The ligand binding mode is the same in A and B, while the conformation assumed by the residues forming the binding cavity, the A-loop and *α*G-helix is different (SI Fig. S8). In B, the A-loop assumes a more open conformation loosing the contact with the *β*_3_-*α*C loop. The loss of this interaction results in a substantial hinge motion, leading to the further opening of the cleft corresponding to the binding site. This opening is necessary to the unbinding of Imatinib. Not only the most probable exit path on the Free Energy Surface (FES) involves an opening of the cleft, but when we tried to compute the unbinding free energy profile without taking explicitly into consideration the hinge motion it leads to unrealistically high energy barriers. The presence of different A-loop conformations in the bound state is consistent with the observed broadening and absence of the NMR signals corresponding to this region.

The salt bridges formed by the four functionally important charged residues (Lys_295_, Glu_310_, Asp_404_ and Arg_409_) coordinate the movements of the P-loop, the A-loop and the *α*C helix (SI Fig. S9), as reported in other kinases^39^,^40^, and allosterically determine the orientation of the *α*G helix and the formation of an additional helix turn in the *α*F helix (SI Fig. S10).

Beyond the first transition state, which corresponds to S_*path*_ ~ 12, an additional metastable minimum is found for values of S_*path*_ around 15–16 (structure C in Fig. 4). In this conformation, Imatinib has moved away from its canonical binding pose and occupies a secondary pocket (sometimes referred to as the “deep pocket”) beneath the *α*C helix in its entirety (SI Fig. S13). The free energy of this secondary minimum is ~4 *kcal*/*mol* higher than the main minimum, suggesting a population around 1%. While the presence of a second binding pose is not directly confirmed by NMR, all the residues that are predicted to interact with Imatinib in this second free energy minimum (SI Fig. S13) also change their dynamical behaviour, as indicated by the appearance of their amide signal in the Imatinib-bound NMR spectrum. NMR measurements performed immediately after addition of the drug tend to exclude a lifetime longer than minutes.

A transient occupation of this external cavity facilitates the entrance of the drug kinetically, at the very beginning of the recognition process and lowers the free energy penalty of the DFG flip (see below). Recent experiments on Src and Abl^22^ have suggested a two step binding process, in which a fast binding event is followed by a slower conformational rearrangement. While the authors in a later paper^23^ interpreted the results as suggestive of a fast binding (in the crystallographic pose) followed by an induced-fit mechanism involving the P-loop, their data are also explained by our model, which is based both on experimental and computational evidence. Our simulations show that the first step is the binding to an external site. The external site has been observed in other kinases and is supported by our NMR data. Surprisingly, Imatinib is able to approach the external binding site irrespective of the DFG conformation. The first binding is followed by a slower “sliding” into the final pose. This latter step involves the drug passing over the DFG and reaching the ATP binding site below the P-loop and both a “conformational selection” (red line 4) or “induced fit” (yellow line) mechanisms are possible. The main association path (as indicated by the free energy surface) involves a conformational selection mechanism, whereby the ligand directly binds to a DFG-out state.

The induced-fit mechanism is less probable than the conformational selection mechanism due to an extra energy barrier (yellow line, 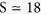) that has to be overcome along this alternative association pathway. Clearly, mutations of the P-loop (including the“gate-keeper” residue) used to indirectly support the P-loop-based induced-fit model^23^, affect the sliding step^25^ and might favour the induced-fit mechanism over the conformational selection.

The new TS corresponds to structures in which Imatinib is in contact with the Phe_405_ (see SI). The absolute binding free energy, obtained by integrating the free energy of the bound and unbound basin^41^, is around −8 *kcal*/*mol* for the “induced fit” path. In the case of the “conformational selection” path (in red), one has to add to the −14 *kcal*/*mol* the Δ*G* necessary for the DFG-flip, which is around 6.0 *kcal*/*mol*^15^,^21^, leading again to a total binding Δ*G* of around −8 *kcal*/*mol*. These values have to be increased by the standard volume correction, (amounting to 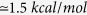) and possibly slightly decreased by change in the protonation state of Imatinib^21^. The final value of −7 ± 1 *kcal*/*mol* is in striking agreement with the experimental value of −6.8 *kcal*/*mol* obtained from the *K*_*d*_. The error bar takes into account only the uncertainty on the reconstructed free energy profiles during the final 100 ns of the PT-metaD sampling. Along the conformational selection mechanism, as soon as the ligand comes in contact with the P-loop of the kinase, the DFG-out state is stabilized (red line). By comparing the free energy difference associated with the DFG-flip at S = 25 with that obtained for the apo-form^15^, we observe that when Imatinib is in contact with the kinase but not directly with the DFG motif the free energy penalty for the flip is decreased of 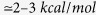 (Fig. 4).

Our proposed model is not only in agreement with the data recently reported^22^,^23^, but it also recapitulates a plethora of previous computational and experimental evidence^10^,^15^,^21^ and it is in substantial agreement with the binding mechanism proposed for a different DFG-out inhibitors binding to the FGFR1 TK^14^.

### Unbinding kinetics and residence time

We measured the dissociation kinetics by surface plasmon resonance (SPR) obtaining at pH 7.4 a dissociation rate between 0.024 and 0.19 *s*^−1^ (SI Fig. S14). The main unbinding barrier, which corresponds to S_*path*_ ~ 12, is predicted by PT-metaD to be 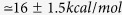. The higher sampling error bar is due to the nature of the PT-metaD algorithm, that samples high-in-energy TS worse than the energy minima. Given the importance of getting a barrier height as accurate as possible, we also recomputed the unbinding barrier with a different algorithm (multiple-walkers^42^), and obtained a value that is well within the error bar of the previous estimate (16.5 *kcal*/*mol*). Given the complex nature of the unbinding mechanism, the standard transition state theory is not applicable here, i.e. we expect that the transmission coefficient is much less than 1. The unbinding rate was instead obtained by using the accurate but computationally expensive TS-PPTIS algorithm^43^, that combines the estimate of the unbinding ΔG with a transmission coefficient computed independently. More than 10000 unbiased MD trajectories where initiated from different interfaces at the barrier region. As expected for a complex diffusive process, the transmission coefficient is much smaller than 1, leading to a dissociation rate of 0.0114 *s*^−1^ (1E-3 to 0.139 *s*^−1^, when the sampling error is accounted for). The predicted range is in excellent agreement with the experimental one.

A representative structure of the transition state (TS) is reported in SI Fig. S8. Imatinib detaches from the hinge region of c-Src, moves closer to the DFG motif and a layer of water molecules intercalates between the compound and the hinge (SI Fig. S11). The most relevant differences are observed for the *α*G helix, which partially unfolds at the TS (SI Fig. S8). This seemingly surprising observation confirms the “cracking” hypothesis, whereby relevant conformational transitions are accompanied by local unfolding^26^. The “cracking” lowers the TS energy by increasing the conformational entropy, as reported in the case of the Epidermal Growth Factor Receptor kinase^44^.

In the case of c-Src, the observed chemical shift indexes for the *α*C and *α*D helices also suggest partial unfolding (SI Fig. S12). Remarkably, the effect of local unfolding on the transition state energy has been recently shown for the binding of the type-II (DFG-out) drug Ponatinib to the Fibroblast Growth Factor Receptor^14^. The higher entropy attained by the “cracking” is needed to overcome the enthalpic penalty due to the DFG flip, as also reported in the case of Abl^25^.

### H/D exchange NMR experiments

**Figure 5:**
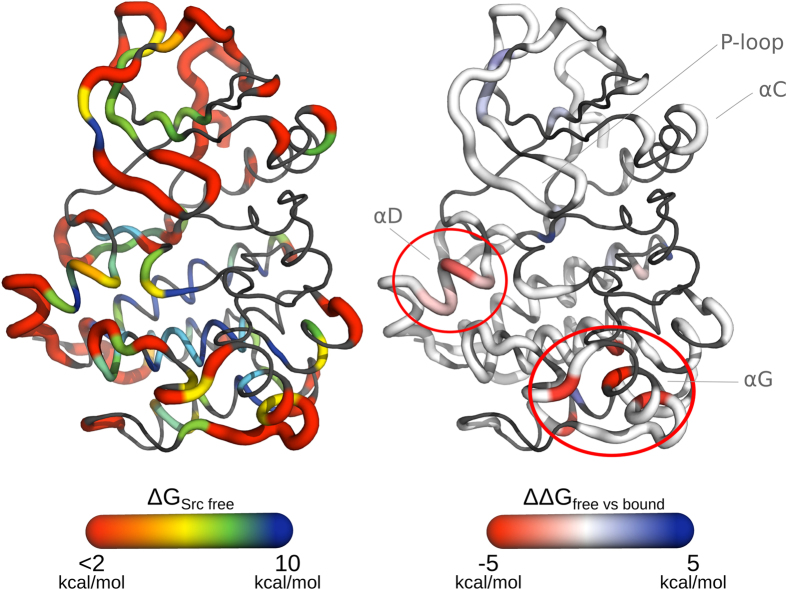
Free energies for local unfolding. The values are derived from the *k*_*intr*_ and *k*_*exch*_ parameters obtained from H/D exchange NMR measurements. Red circles indicate *α*D and *α*G, the regions with increased exposure to the solvent in the bound structure.

To investigate the dynamics on longer time scales and, possibly, further confirm that a cracking-like mechanism is involved, we performed H/D exchange NMR experiments and determined the protection factors and free energies for local unfolding^45^ (Fig. 5, SI Methods and SI Fig. S7). The values are directly related to the ease of exchange of amide protons with the solvent and give a good estimate of the energy needed to locally unfold the structure. Lower values correspond to regions that are (at least transiently) solvent exposed and unfolded.

While the C-lobe in both the apo and Imatinib-bound form is mostly folded and rigid, the *α*D, *α*G and part of the *α*I helices are a notable exception. Their lower protection factors indicate higher flexibility, in agreement with the chemical shift indexes. In presence of Imatinib these helices have an even lower protection factor, consistent with the transient unfolding of the *α*G helix observed in the simulations. As Imatinib is a xenobiotic compound, it remains an open question whether “cracking” is also involved in the physiological binding and release of ATP/ADP. The change in the flexibility of the *α*I helix is also intriguing, as this structural motif has been recently shown to play a key role in the interaction with the SH2 regulatory domain^46^.

## Discussion

The catalytic domain of c-Src assumes a compact and relatively rigid structure in solution. With the exception of the N-terminus (whose biological significance is limited in a truncated protein), loops are only slightly more flexible than the elements of secondary structure (in the ps-ns time scales). In the apo form the *α*C-helix, the A-loop and other functionally relevant motifs undergo significant conformational changes on time scales spanning the *μ*s-ms range. MD simulations show different conformations of the *α*C helix, in agreement with the *α*C-in and *α*C-out conformations captured in different crystal structures^35^.

The conformational landscape of the *α*C-helix is altered by the binding of Imatinib, as proven by the computed free energy landscape and the appearance of most missing signals in the NMR spectra. On the contrary, the signals corresponding to the A-loop are not visible neither in the spectra of the apo nor of the ligand-bound forms due to conformational heterogeneity. However, the conformations spanned by the apo and bound forms are different. Simulations^18^,^19^ and crystal structures^47^,^48^ hint to an equilibrium between the active and inactive forms.

When Imatinib is bound, steric hindrance prevents the A-loop from assuming a fully extended active form. Indeed, the simulations predict an equilibrium between closed and semi-closed A-loop conformations.

The computed binding ΔG is in good agreement with the experiment. The free energy calculations of the DFG-in to DFG-out conformational change in presence of Imatinib show that Imatinib can approach the site irrespective of the DFG conformation. The main association path involves a conformational selection, with a small induced-fit effect, predicted to be 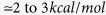. The balance between the two might change at different temperatures due to the different entropic/enthalpic balance.

The induced fit effect is also in agreement with the presence of a second “external” binding pose clearly visible in the binding free energy profile and suggested by the change of dynamics of the entire region encompassing the binding pathway. A two step binding mode involving an external pose has also been suggested in the literature for other PKs^49^,^50^ and is consistent with recent NMR experiments^22^.

Concluding our description of c-Src dynamics is the surprising flexibility of helix *α*G. This helix, together with the flanking loops, is the only one experiencing relevant dynamics in the sub-*μ*s time scale. Over longer time scales, the *α*G appears to correlate with the motion of the A-loop, assuming different orientations as a consequence of its conformational rearrangements, and plays an important role in the binding of Imatinib. What is more, the *α*G helix appears to unfold (“cracking”) at the transition state. The excellent agreement between the predicted *k*_*off*_ with that measured with SPR as well as the changes in the dynamics observed by H/D exchange NMR experiments, confirm the role of the *α*G cracking, providing further evidence for the “cracking” mechanism, which was also reported in other kinases^14^,^44^.

Taken together, our experimental and in-silico results give a solid and comprehensive picture of the mechanism of binding of the anti-cancer drug Imatinib to c-Src and the ensuing local and long-range dynamics. Both local and global conformational changes and transient unfolding impact the overall kinase behavior in response to molecular recognition. This detailed information is of great importance to reconcile opposing view held in the literature and might help the rational development of Src inhibitors. Indeed, the long-range allosteric effect described here, linking the *α*G to the active site, might be used for the development of innovative allosteric inhibitors of this and other tyrosine kinases^51^.

## Methods Summary

The molecular dynamics simulations were performed with ACEMD^52^, GROMACS^53^ and the Amber99SB*-ILDN^54^,^55^ force field. Imatinib was parametrized with GAFF^56^ on the basis of *ab-initio* calculations. All free energy calculations were performed with PT-metaD using GROMACS^53^ and the PLUMED plugin^57^. NMR experiments were performed on Bruker Avance 600 MHz, 700 MHz and 1 GHz, the latter equipped with cryogenically-cooled triple resonance probe. Protein samples were prepared as in ref. 30 and solvated in 20 mM phosphate at pH 6.4, 0.5 M NaCl, 1.0 mM MgCl_2_, 1 mM TCEP and 0.03% NaN_3_. NMR Relaxation experiments were performed at 700 MHz on a ^15^N labelled sample. All SPR measurements were performed with a Biacore X (GE Healthcare) system. (Full methods in SI).

## Additional Information

**How to cite this article**: Morando, M. A. *et al*. Conformational Selection and Induced Fit Mechanisms in the Binding of an Anticancer Drug to the c-Src Kinase. *Sci. Rep*. **6**, 24439; doi: 10.1038/srep24439 (2016).

## Supplementary Material

Supplementary Information
