# Supplementary material for: Conformational Selection and Induced Fit Mechanisms in the Binding of an Anticancer Drug to the c-Src Kinase
Source: Sci Rep. 2016 Apr 18;6:24439. doi: 10.1038/srep24439 (PMC4834493; doi:10.1038/srep24439)
Supplement: Supplementary Information [file srep24439-s1.pdf]

Supplementary Information for

Conformational Selection and Induced Fit Mechanisms in the Binding of an Anticancer Drug to the c-Src Kinase

Maria Morando,[1,*] Giorgio Saladino,[2,*] Nicola D'Amelio,[2] Encarna Pucheta-Martinez,[3] Silvia Lovera,[3] Moreno Lelli,[4] Marco Marenchino,[5] Blanca López,[5] Ramón Campos-Olivas,[5] and Francesco Luigi Gervasio[2,3,†]

[1] *Structural Biology and Biocomputing Programme, Spanish National Cancer Research Centre (CNIO), c/ Melchor Fernandez Almagro 3, 28029, Madrid, Spain*

[2] *Institute of Structural and Molecular Biology, University College London, London WC1E 6BT, United Kingdom*

[3] *Department of Chemistry, University College London, London WC1E 6BT, United Kingdom*

[4] *Centre de RMN à Très Hauts Champs, Institut de Sciences Analytiques, (CNRS/ENS Lyon/Universitè CB Lyon 1), 69100 Villeurbanne, France*

[5] *Spectroscopy and NMR Unit, Spanish National Cancer Research Centre (CNIO), c/ Melchor Fernandez Almagro 3, 28029, Madrid, Spain*

* These authors contributed equally.

† f.l.gervasio@ucl.ac.uk

	600 MHz	700 MHz	1 GHz
$R1^{calc}$	0.54 ± 0.03	0.43 ± 0.02	0.28 ± 0.01
$R1^{exp}$	0.61 ± 0.11	0.44 ± 0.06	0.39 ± 0.11
$R2^{calc}$	35.5 ± 1.7	38.8 ± 2.0	51.4 ± 2.6
$R2^{exp}$	43.0 ± 8.7	44.1 ± 6.5	50.0 ± 9.4

Table I. Average values and standard deviations of R1 and R2 values as calculated by hydronmr [1] using the crystal structure of c-Src catalytic domain (PDB ID: 2SRC). Values are compared with experimental values. In both cases averages are calculated excluding the flexible N terminal tail comprising residues 251-263.

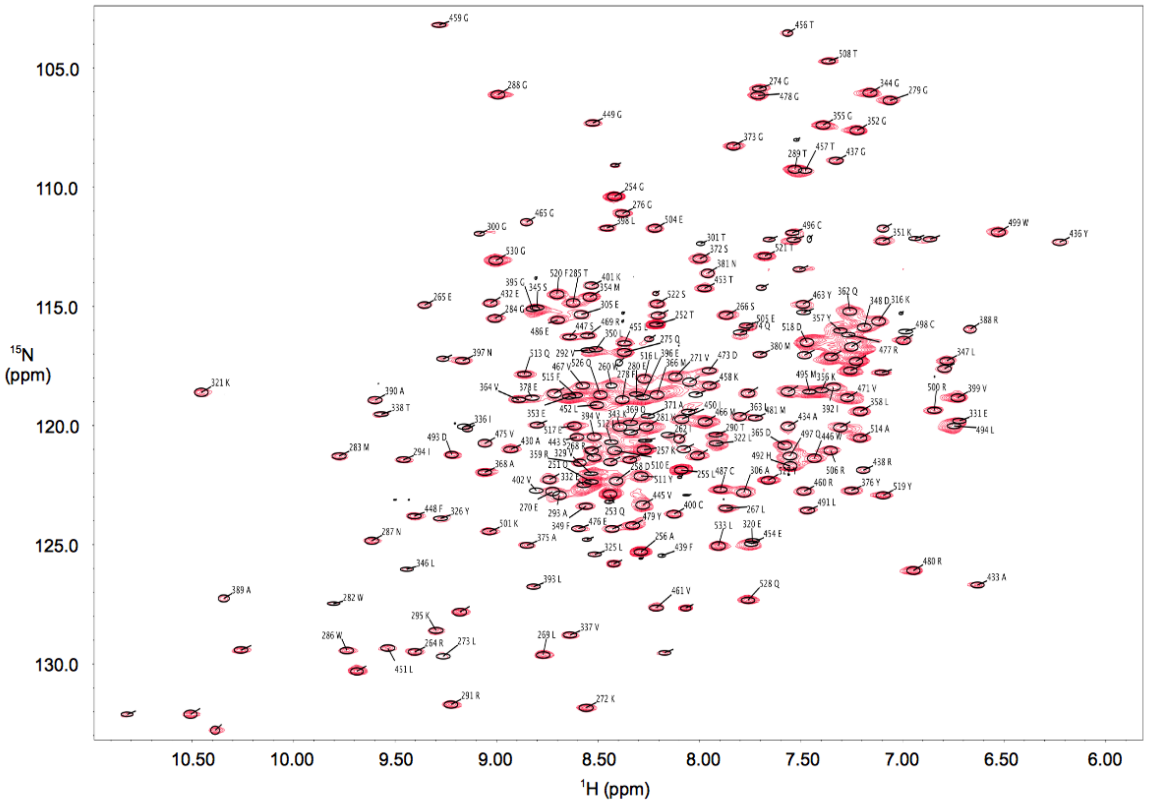

Figure S1. NMR ^{1}H,^{15}N-TROSY spectrum of c-Src catalytic domain at pH 6.4, 293 K

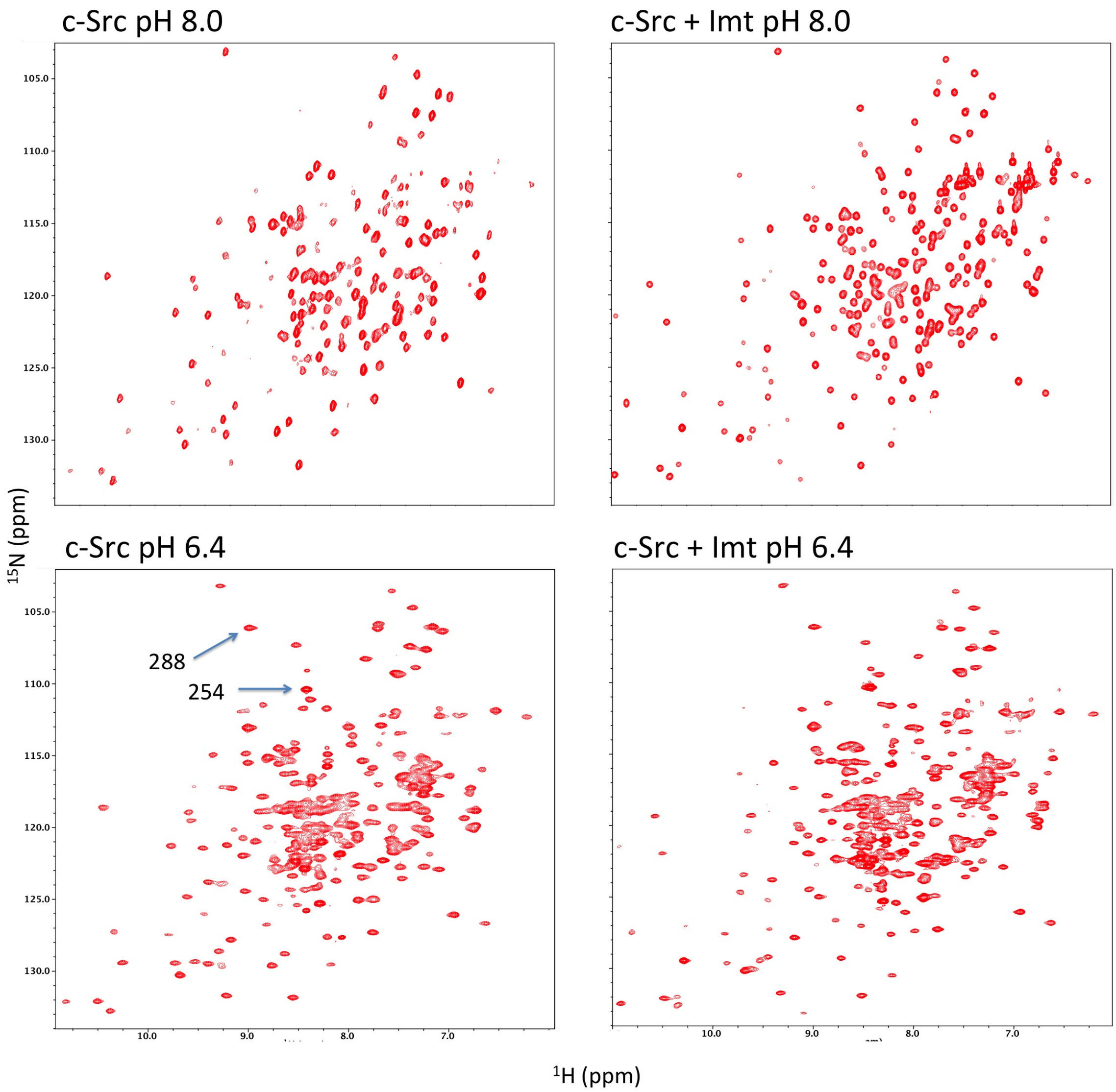

Figure S2. 2D ^{1}H,^{15}N-TROSY NMR spectra of c-Src catalytic domain in the absence (left) and in the presence (right) of Imatinib at pH 8.0 (top) and 6.4 (bottom). At lower pH signals of residues exposed to the solvent appear. Two examples are highlighted referring to the N terminus (residue 254) and the β_2-β_3 loop (residue 288).

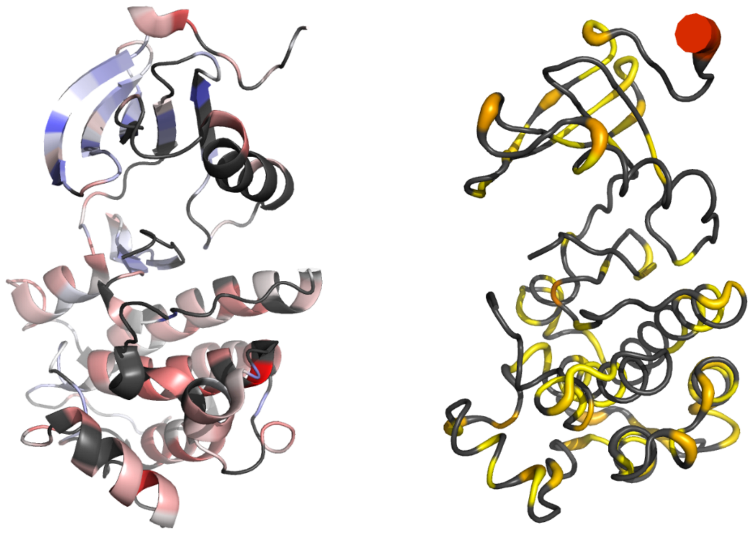

Figure S3. (left) Chemical shift deviations from random coil values of carbonyl carbons in free Src. Positive and negative deviations (indicating tendency to alpha and beta structures respectively) are displayed in red and blue. (right) Order parameter S^2. Values decrease from yellow to red (the information is further displayed by the thickness of the backbone which is inversely proportional to the degree of order). In both panels residues for which data is not available are displayed in gray.

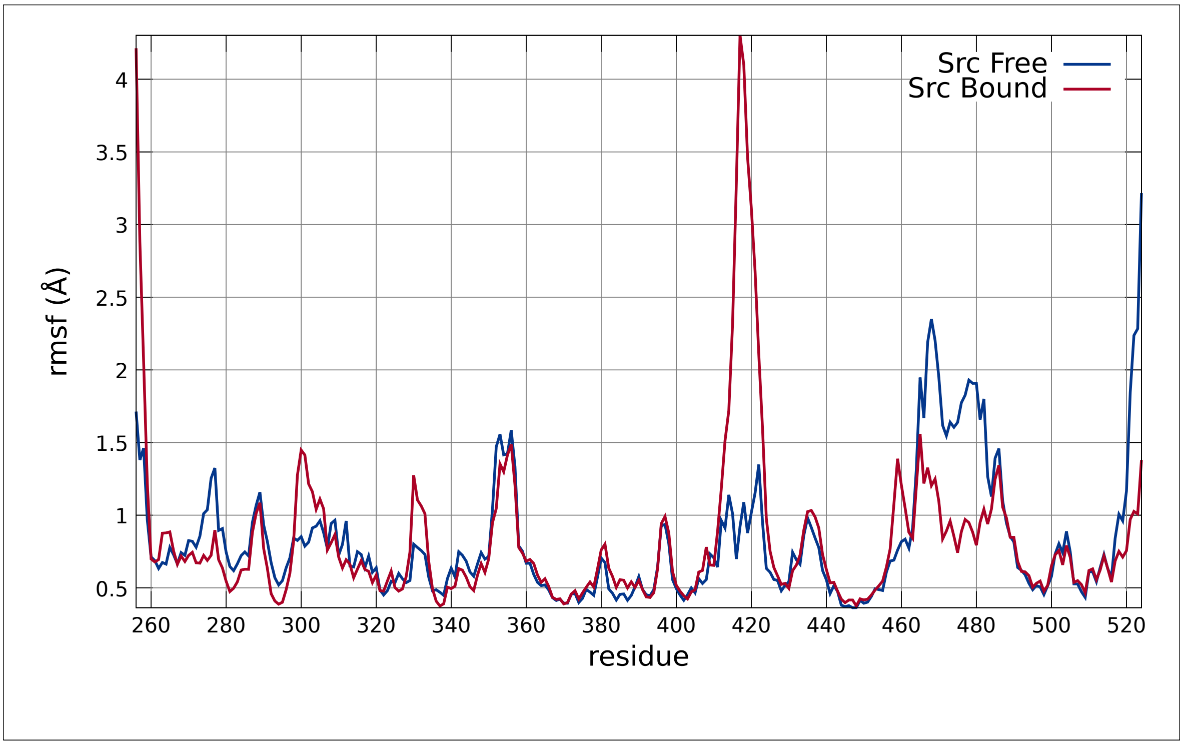

Figure S4. Average C_α root mean square fluctuations (RMSF) for the apo and Imatinib-bound structures of c-Src as obtained from a 1 μs-long MD simulation.

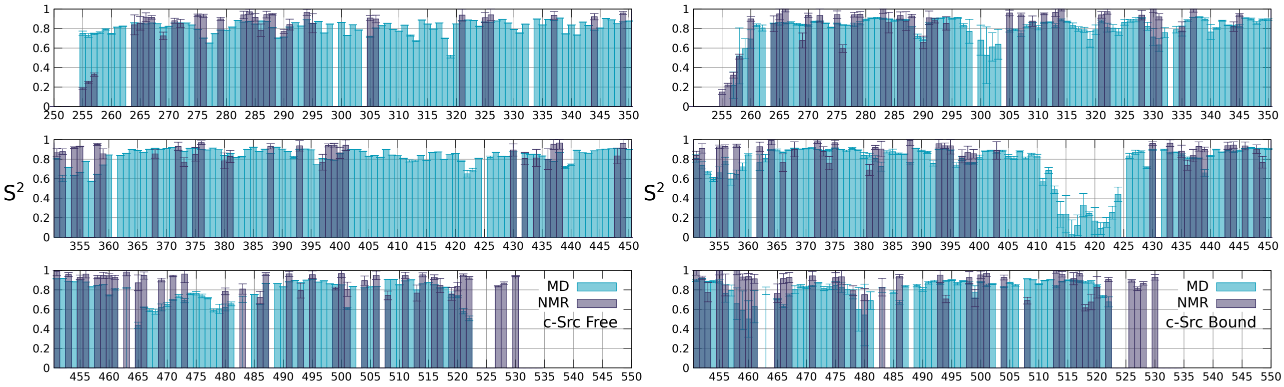

Figure S5. Order parameters S^2 calculated from the NH vector rotational correlation functions of the simulations of c-Src Free (left) and Bound (right) compared to the experimental results.

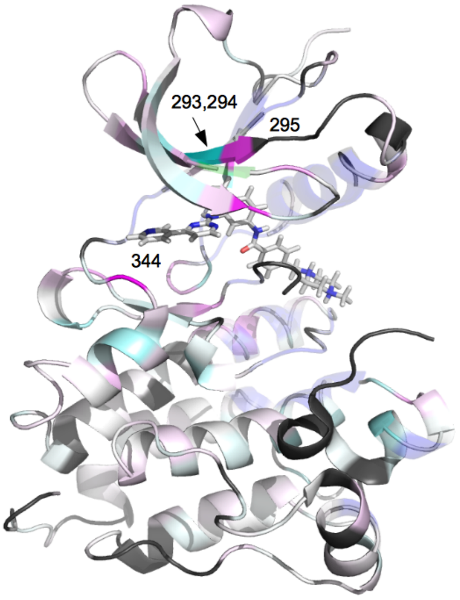

Figure S6. ^{1}H chemical shift deviations upon Imatinib binding. Positive and negative deviations (deshielding and shielding) are highlighted in magenta and cyan, respectively. Residues appearing and disappearing in the ^{1}H, ^{15}N-TROSY spectrum upon addition of Imatinib are shown as transparent (blue and green, respectively). Residues for which data is not available are displayed in grey, while those experiencing large deviations are indicated onto the structure.

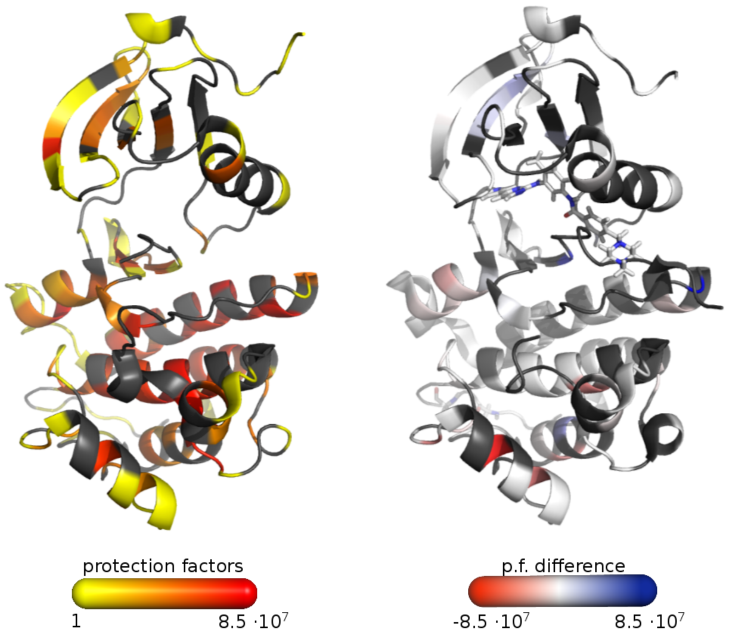

Figure S7. (left) Protection factors mapped onto the crystal structure of Src kinase domain (pdb code 2OIQ). Values increase from yellow to red. (right) differences in the value of protection factors in the presence of Imatinib. Decrease and increase in protection are highlighted in red and blue, respectively. In both panels residues for which data are not available are displayed in gray.

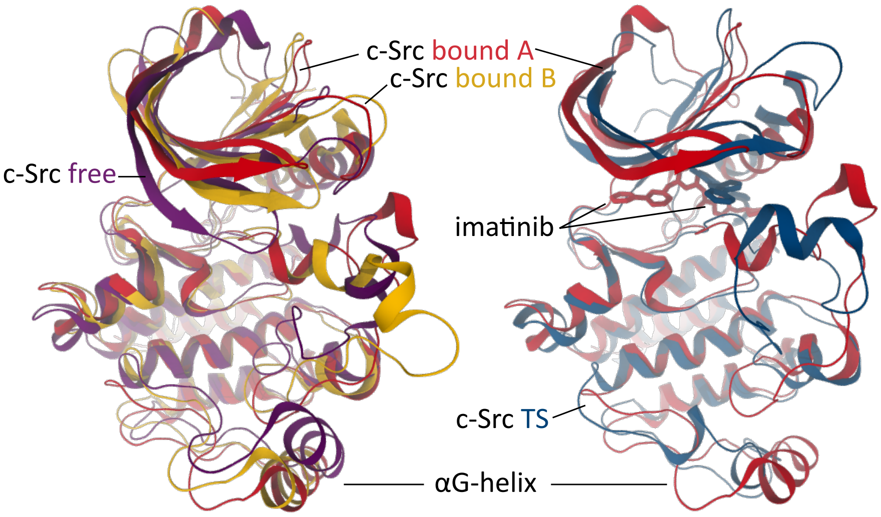

Figure S8. Conformations of c-Src: (left) the free structure is compared with the structures (bound A, B) extracted from the two main minima of the free energy landscape, (right) conformation of the transition state compared to the main FE minimum. A partial unfolding of helix αG is observed.

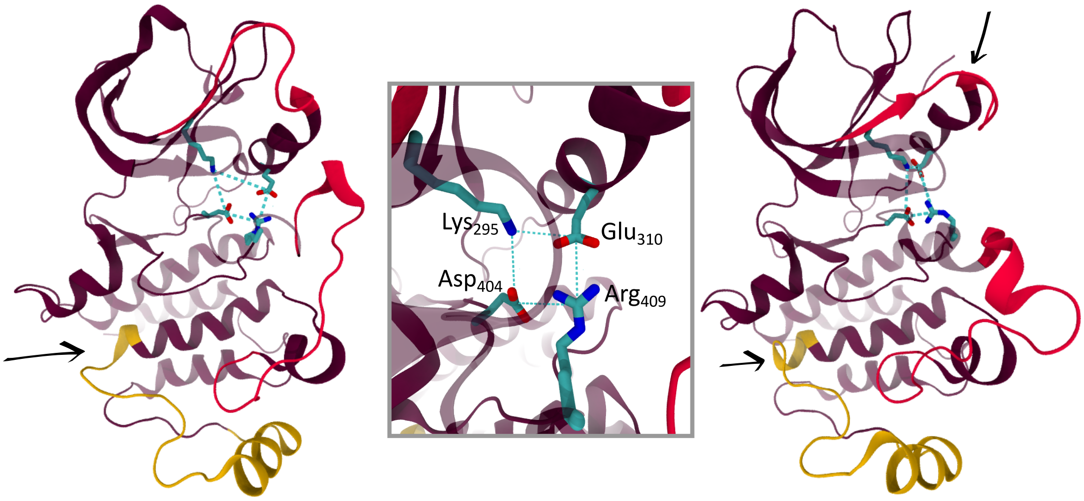

Figure S9. Configurations of the salt bridges in the conformations of c-Src bound to Imatinib (A,B in Figure S8). Structural elements correlated with the salt-bridge configuration are colored in red ($\beta_3 - \alpha$C loop and A-loop) and yellow (αF and αG). Black arrows indicate the formation of an additional helix turn in αF in structure B.

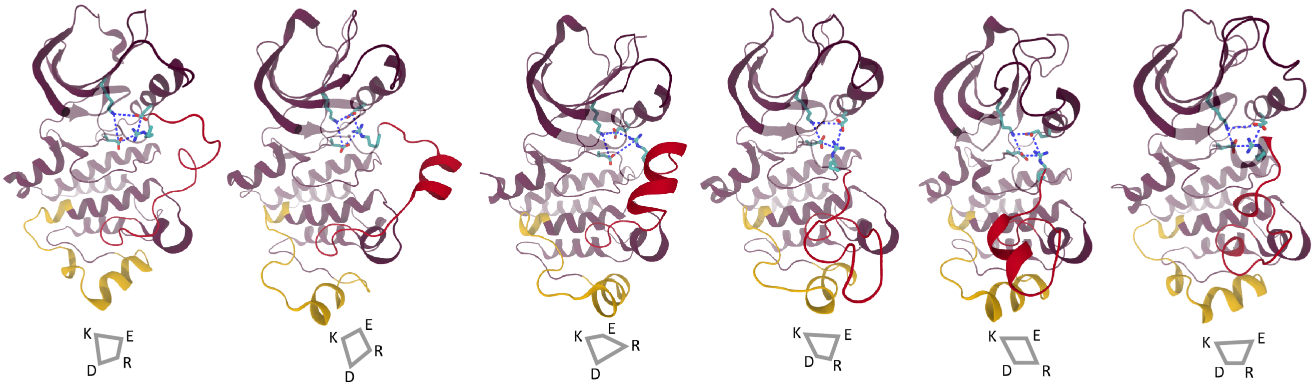

Figure S10. A subset of representative secondary, less populated, conformations of c-Src bound to Imatinib as observed throughout the simulation. The configuration of the important salt-bridges (indicated with grey trapezoids) is correlated with the conformations of the A-loop (in red) and αF and αG (in yellow).

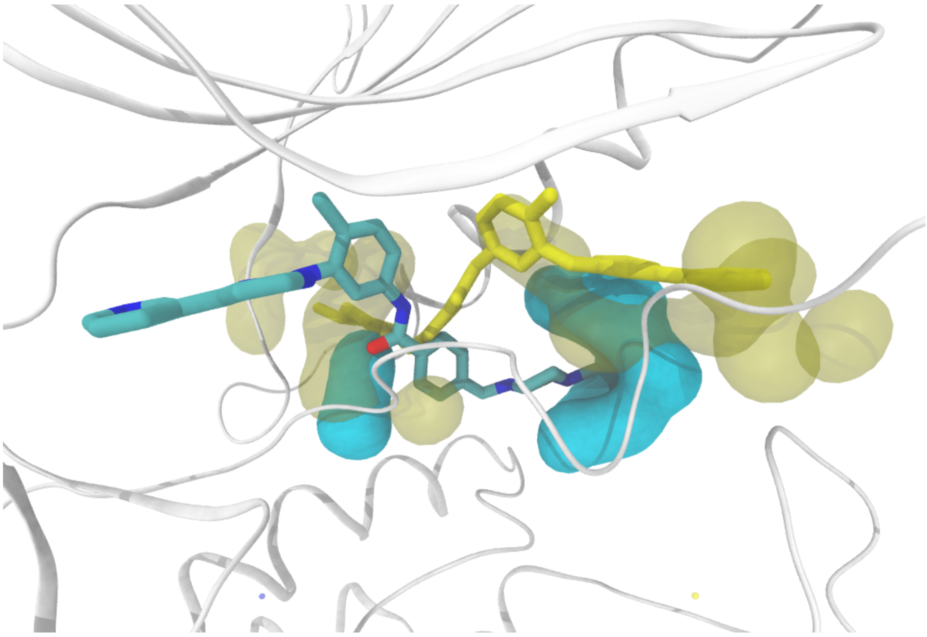

Figure S11. Crystallographic (blue) and ”external” (yellow) binding pose of Imatinib. The ligand is represented as sticks while the first hydration shell as transparent isosurface. The main difference is found at the interface with the hinge region where only the external pose is hydrated.

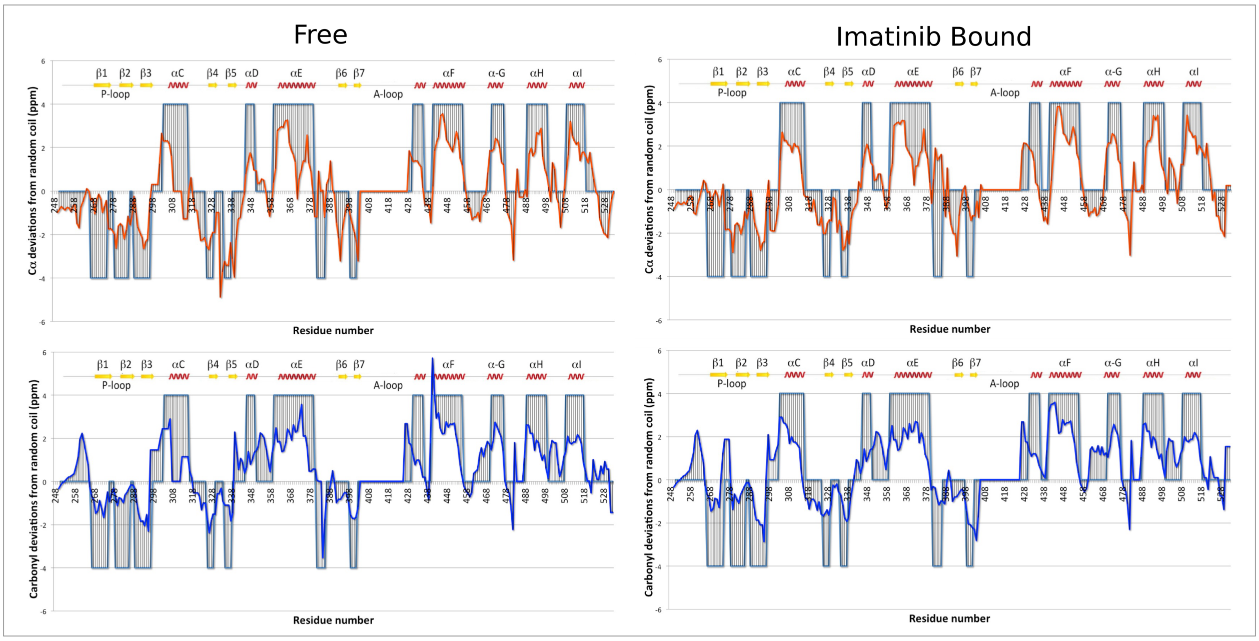

Figure S12. Carbonyl (blue) and C_α (red) chemical shift deviations from random coil values measured for the catalytic domain of c-Src. The figure reports centered running average over 5 residues. Elements of secondary structure found in the crystal (pdb 2OIQ) are schematically displayed and an ideal deviation of 4 ppm is reported as a continuous shadowed area (positive for alpha helices, negative for beta strands)

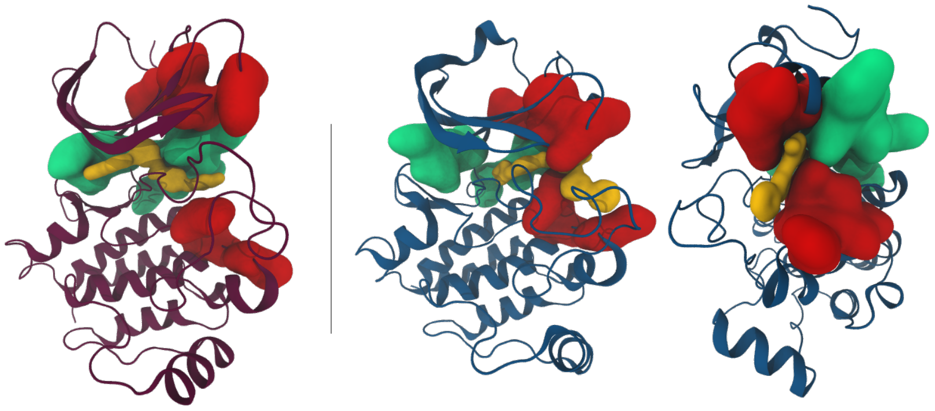

Figure S13. Residues appearing in the bound NMR spectra and belonging to the main (green) or secondary (red) binding site as represented on top of the canonical bound structure (left, purple, corresponding to structure A in Figure 4) and of the secondary bound structure (right, blue, structure C in Figure 4). The residues highlighted in red are far apart in the bound structure and come closer to outline the secondary binding cavity in the secondary bound pose.

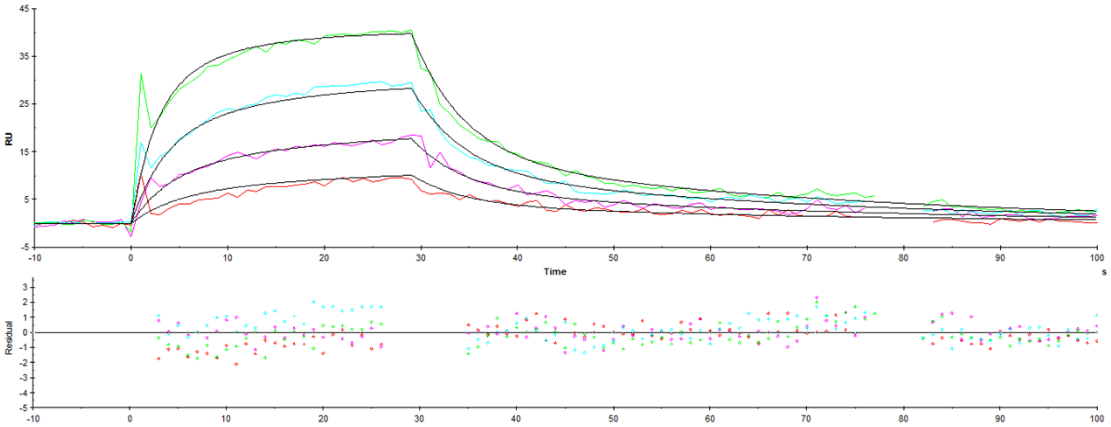

Figure S14. Surface Plasmon Resonance spectra of imatinib binding to Src WT at 20 °C.

MATERIAL AND METHODS

Molecular Dynamics Simulations

The kinases structures were retrieved from the Protein Data Bank (PDB entries 2SRC and 2OIQ). Missing residues in 2OIQ were added using the software Modeller [2], according to the respective Uniprot sequence and using 2SRC as a template. For the apo structure we used the Amber99SB*-ILDN [3, 4] force field, including backbone corrections by Hummer and Best [5] with explicit TIP3P [6] water molecules. For the holo structure we used Amber99SB*-ILDN [3, 4] force field for the protein part and GAFF [7] for the ligand. The ligand charges were calculated at the HF level using a 631-G* basis set with the Gaussian03 [8] package. Preliminary simulations indicated that the default GAFF parameters for the dihedrals $ca - ca - n - c$ and $ca - ca - c3 - n3$ didn't reproduce the correct torsional profile. For this reason, QM-level torsional scans with a step of 10 degrees were carried out for these dihedrals. Torsional profiles with the GAFF force field in vacuum were then fitted to the QM ones in order to derive more accurate parameters. All the systems were minimized with 10000 steps of conjugated gradient and equilibrated in the isothermal-isobaric (NPT) ensemble for 10 ns, using a Berendsen barostat to keep the pressure at 1 atm. The temperature was kept at 305 K by a Langevin thermostat. A 1 μs production run was carried out for all the systems in the canonical (NVT) ensemble. All the simulations were carried out with the ACEMD program [9] running on a server with four Nvidia Fermi GPUs. The van der Waals interactions were cut off at 1.0 nm, and long range electrostatic interactions were calculated by the particle mesh Ewald algorithm with a mesh spaced 0.1 nm. These values have been shown to perform optimally on GPUs. The parallel-tempering metadynamics (PT-MetaD) simulations were performed using Gromacs 4.5.5 [10] combined with the PLUMED 1.3 [11] plug-in. The particle mesh Ewald (PME)-Switch algorithm was used for electrostatic interactions with a cut-off of 1 nm. A single cut-off of 1.2 nm was used for Van der Waals interactions. Temperature coupling was done with the V-re-scale algorithm [12]. We performed PT-MetaD with 5 replicas using the Well Tempered Ensemble [13] with 3 Collective Variables (CVs), the 2 Path Collective Variables (PCVs) s and z [14], plus a CV counting the number of water molecules interacting with both the ligand and the cavity. The bias factor was set to 15.0 and the Gaussians height to 1.25 kJ/mol, with a deposition rate of 1/2000 steps. The Gaussian width was set to 0.1, 0.005 and 0.1 for the three CVs, respectively. As customary in the case of ligand binding [15, 16], we performed a preliminary metadynamics run using a simple geometrical CV to obtain an initial pathway for the setup of the PCVs. We selected 27 frames from the lowest energy path obtained in the preliminary run and optimized this initial guess using the methodology described by Branduardi et al.[14]. As the preliminary metadynamics showed large rearrangements of the A-loop, we included Cα atoms of the loop and of the αC-helix in the definition of the PCVs. Two extra CVs defined as the distance between Asp404 and Lys295 and the distance between Phe405 and Leu317 (as in Lovera et al. 2012) were used to describe the DFG-flip. The sampling convergence was checked by comparing the reconstructed free energy surfaces at different time intervals during the last 50 ns of the simulations. As the sampling on top of the rate-determining TS was less exhaustive than in the main minima, we also performed Multiple-Walkers metadynamics using 5 walkers starting from the bound state and the same setup of the PTMetaD run. The value of the multiple-walkers determined tranistion state energy is within the error bar of the PTmetaD one.

In order to assess the agreement between the simulations and the dynamics observed by NMR, we used the MD trajectories to calculate the order parameter S^2 from the rotational correlation function of the backbone amide vector, according to Lipari and Szabo. In our case, in the context of an extended model [17], we used the more general expression introduced by Bremi et al. [18], according to which the internal motion correlation function is fitted with a sum of five exponentials

$$C_I(t) = A_0 + \sum_{k=1}^{5} A_k \cdot e^{-\frac{t}{\tau_k}}$$

where A_0 is analogous to the order parameter S^2, while the other $A_{k>0}$ are indicative of motions on different (slower) time-scales. The internal NH motion was decoupled from the overall motion by rotational and translational fitting to a reference structure. As for the RMSF, the analysis was repeated in windows to assess the accuracy of the estimate.

NMR experiments

Sample preparation

Expression and purification were performed following the procedure published elsewhere [19]. Protein concentrations were determined by absorbance spectroscopy using a NanoDrop ND -1000 (ϵ_{280} =52370 M^{-1} cm^{-1}). Stock solutions

of the proteins were stored at 4 °C, for short-term use, or at -20 °C.

NMR assignment

Protein samples were prepared in 20 mM sodium phosphate at pH 6.4, 0.5 M NaCl, 1mM MgCl2, 1mM TCEP, and 0.03% NaN_3. The temperature was set to 293 K. The choice of the pH was dictated by a compromise between protein solubility (decreasing at lower pH) and the detectability of water-exchanging amide protons. NMR experiments were performed on Bruker Avance 600 MHz, 700 MHz and 1 GHz, the latter equipped with cryogenically-cooled triple resonance probe. Sample concentration was 300 μM. ^{1}H and ^{15}N backbone assignment of the catalytic domain of Src was performed using a series of three-dimensional experiments recorded at both 700 MHz and 1 GHz, namely: trHNCO , trHN(CA)CO, trHNCA, trHNCACB and ^{1}H,^{15}N -NOESY-HSQC recorded on a doubly or triply labeled sample with deuteration > 70%. The assignment was aided by the comparison with the assignment of the Src-Imatinib complex [19], corrected for the different pH by a titration from pH 8 to 6.4. Chemical shift values were further checked by comparison with predictions from the crystallographic structure and random coil using SPARTA+ [20] and ncIDP programs [21]. Spectra were processed with XWINNMR 2.1 program and analysed with NMRViewJ (One Moon Scientific) [22]. Carbon Ca and Cb chemical shifts were corrected for the deuterium isotope effect [23]. Chemical shift Index was calculated for Cα , Cβ and carbonyl chemical shifts as described by Wishart et al. [**?**]

NMR Relaxation

Relaxation experiments for the mobility measurements of free c-Src were performed at 700 and 1000 MHz using pulse sequences for ^{15}N R_1 and R_2 rates, and heteronuclear NOE spectra as described in the literature [24–29]. Relaxation delays for T_1 were: 50, 200, 400, 600, 1000, 1500, 2000, 3000 ms while for T2 measurements we used the following delays: 0.0, 6.3, 12.6, 18.9, 25.2, 31.4 and 37.7 ms. Due to the short transversal relaxation times the pulse sequence was modified in order to minimize the CPMG delay to 6 ms. This allowed to sample more time points in T_2 decays. Measurements were repeated in the presence of Imatinib (1.5 equivalents) at 700 MHz. Addition of Imatinib in ratio protein:drug 1: 1.5 was achieved adding few micro-liters of a concentrated (typically 50 mM) DMSO solution. TENSOR2 [30], was used to analyze the data, yielding the isotropic rotational correlation time τ_c and the Lipari-Szabo sub-nanosecond order parameters (S^2) [31].

The isotropic rotational correlation time τ_c estimated from the R2/R1 ratio for a solution 300 μM of free c-Src at 700 MHz is slightly larger than expected (26 ns). This is probably due to the onset of aggregation, as in the case of Abl [32]. When we repeated the analysis on the data obtained on a 1GHz spectrometer on a less concentrated sample (< 100 μM) , it resulted in a value for τ_c of 21 ns, which is close to the value expected for a monomeric protein of this size [33] and in excellent agreement with the value estimated by Hydronmr [1] using the crystallographic c-Src PDB structure (2OIQ).

However, at the lower concentration the signal to noise ratio was too low to estimate the S^2 for many of the observed peaks, while for the well resolved and intense peaks, the estimated S^2 is in good agreement with that obtained at 700MHz on the more concentrated solution. Indeed, the chemical shifts of the protein remain unaltered up to concentrations of 600 μM, a further indication that the onset of aggregation phenomena at 300 μM should have limited effects on the structure and local dynamics of the protein.

Thus, we used the relaxation data obtained with a concentration of 300 μM at 700 MHz to estimate the order parameter S^2. Due to the large number of residues with exchange contributions to R2 (large R1R2 products) or fast mobility (low heteronuclear NOE values), a spherical model was used for the rotational diffusion tensor, as in the case of Abl kinase [32].

Deuterium exchange measurements

H/D exchange was measured following the disappearance of peaks in the ^{1}H,^{15}N - HSQC spectrum. 2D HSQC spectra were recorded at increasing times after resuspension of the lyophilized protein in D_2O-based buffer (20 mM phosphate at pD 6.4, NaCl 0.5 M, MgCl2 1mM, TCEP 1mM, NaN3 0.03%.), extending to a final exchange time of 52.5 days (1260 hours). A total of 26 2D spectra were recorded for each of the two different samples (in the presence and in the absence of Imatinib), with variable number of scans (increasing from 64 in the first time-point to 1024 for the last ones), according to which intensities were also corrected for quantification of exchange rates. Exchange rates were estimated fitting peak intensities as a function of time by regression analysis with a three parameters exponential curve of the type Ae^{-Bt} + C, where B is the exchange rate k_{exch}. Uncertainties in the exchange rates were obtained

from 250 MonteCarlo simulations at a confidence interval of 90% and using the standard deviation of the spectral noise to account for intensity uncertainties. Protection factors (pf) were estimated as the ratio k_{intr}/k_{exch} [34, 35] where k_{intr} is the intrinsic exchange rate calculated from the primary sequence using the SPHERE program [34] and k_{exch} is the experimental value. Protection factors were converted in free energies of local folding in the assumption of EX2 regime [36] (where both the intrinsic exchange rate and the local unfolding kinetic constants are much larger than the local kinetic constant of folding). Such regime is considered quite common in proteins especially for folded protein below pH 9 [37]. Under this assumption the free energy can be calculated as $RT \ln(pf)$. Peaks already disappeared in the first spectrum after dissolution in D_2O were given an exchange rate equal to k_{intr}. Peaks not decreasing in intensity after 52 days were given an exchange rate of 10^{-4} h^{-1}.

Surface Plasmon Resonance

All measurements were performed with a Biacore X (GE Healthcare) system. The protein was covalently bound to the activated carboxyl groups of the dextran molecules of the Sensor Chip CM5 at 25 °C with running buffer PBS-P (10 mM phosphate buffer, 2.7 mM KCl, 137 mM NaCl, 0.05% P20). The dextran matrix was activated with a solution of EDC/NHS. The protein was diluted to 50 μg/ml in 10 mM sodium acetate pH 5.0 and injected over the surface until the target level of 12300 RU was reached. The remaining activated dextran was inactivated by injection of 1 M ethanolamine. Flow rate was 10 μl/min throughout the immobilization. The cell placed upstream to the active one was left untreated and served as reference cell. The theoretical R_{max} value for the binding of imatinib (MW 494) to Src (MW 35476) was calculated as 170 RU. Kinetic assays were performed at 20 °C in running buffer supplemented with 5% DMSO. Imatinib was diluted into running buffer with 5% DMSO and a serial dilution series was created with a 2-fold dilution factor to give concentrations of 1.56, 3.25, 6.5, and 12.5 μM. The analyte was injected for 30 seconds at 100 μl/min and the dissociation was monitored for 70 seconds. Experimental data were fit with a heterogeneous ligand model, using one global R_{max} parameter, one global k_a parameter, one global k_d parameter and local RI (bulk refractive index) parameters set constant to 0.

---

[1] García de la Torre, J., Huertas, M. L. & Carrasco, B. HYDRONMR: prediction of NMR relaxation of globular proteins from atomic-level structures and hydrodynamic calculations. J. Magn. Reson. **147**, 138–46 (2000).

[2] Sali, A. & Blundell, T. L. Comparative protein modelling by satisfaction of spatial restraints. J. Mol. Biol. **234**, 779–815 (1993).

[3] Hornak, V. et al. Comparison of multiple Amber force fields and development of improved protein backbone parameters. Proteins: Struct. Funct. Bioinf. **65**, 712–725

[4] Lindorff-larsen, K. et al. Improved side-chain torsion potentials for the Amber ff99SB protein force field. Proteins **78**, 1950–8 (2010).

[5] Best, R. B. & Hummer, G. Optimized molecular dynamics force fields applied to the helix-coil transition of polypeptides. J. Phys. Chem. B **113**, 9004–15 (2009).

[6] Jorgensen, W. L. Transferable intermolecular potential functions for water, alcohols, and ethers. Application to liquid water. J. Am. Chem. Soc. **103**, 335–340

[7] Wang, J., Wolf, R. M., Caldwell, J. W., Kollman, P. A. & Case, D. A. Development and testing of a general amber force field. J. Comput. Chem. **26**, 114–114 (2005).

[8] Frisch, M. J. et al. Gaussian 03, Revision C.02 (2003). Gaussian, Inc., Wallingford, CT, 2004.

[9] Harvey, M. J., Giupponi, G. & De Fabritiis, G. ACEMD: Accelerating Biomolecular Dynamics in the Microsecond Time Scale. J. Chem. Theory Comput. **5**, 1632–1639 (2009).

[10] Hess, B., Kutzner, C., van der Spoel, D. & Lindahl, E. GROMACS 4: Algorithms for Highly Efficient, Load-Balanced, and Scalable Molecular Simulation. J. Chem. Theory Comput. **4**, 435–447 (2008).

[11] Bonomi, M. et al. PLUMED: a portable plugin for free-energy calculations with molecular dynamics. Comp. Phys. Comm. **180**, 1961–1972

[12] Bussi, G., Donadio, D. & Parrinello, M. Canonical sampling through velocity rescaling. J. Chem. Phys. **126**, 14101

[13] Bonomi, M. & Parrinello, M. Enhanced Sampling in the Well-Tempered Ensemble. Phys. Rev. Lett. **104**, 1–4 (2010).

[14] Branduardi, D., Gervasio, F. L. & Parrinello, M. From A to B in free energy space. J. Chem. Phys. **126**, 054103 (2007).

[15] Fidelak, J., Juraszek, J., Branduardi, D., Bianciotto, M. & Gervasio, F. L. Free-energy-based methods for binding profile determination in a congeneric series of CDK2 inhibitors. J. Phys. Chem. B **114**, 9516–24 (2010).

[16] Saladino, G., Gauthier, L., Bianciotto, M. & Gervasio, F. L. Assessing the performance of metadynamics and path variables in predicting the binding free energies of p38 inhibitors. J. Chem. Theory Comput. **8**, 1165–1170 (2012).

[17] Clore, G. M. et al. Deviations from the simple two-parameter model-free approach to the interpretation of nitrogen-15 nuclear magnetic relaxation of proteins. J. Am. Chem. Soc. **112**, 4989–4991 (1990).

[18] Bremi, T., Bru, R. & Ernst, R. R. A Protocol for the Interpretation of Side-Chain Dynamics Based on NMR Relaxation : Application to Phenylalanines in Antamanide **7863**, 4272–4284

[19] Campos-Olivas, R., Marenchino, M., Scapozza, L. & Gervasio, F. L. Backbone assignment of the tyrosine kinase Src catalytic domain in complex with imatinib. Biomol. NMR Assign. **5**, 221–4 (2011).

[20] Shen, Y. & Bax, A. SPARTA+: a modest improvement in empirical NMR chemical shift prediction by means of an artificial neural network. J. Biomol. NMR **48**, 13–22 (2010).

[21] Tamiola, K. & Mulder, F. A. A. ncIDP-assign: a SPARKY extension for the effective NMR assignment of intrinsically disordered proteins. Bioinformatics **27**, 1039–40 (2011).

[22] Johnson, B. A. Using NMRView to visualize and analyze the NMR spectra of macromolecules. Methods Mol. Biol. **278**, 313–52 (2004).

[23] Venters, R. A., Farmer, B. T., Fierke, C. A. & Spicer, L. D. Characterizing the use of perdeuteration in NMR studies of large proteins: 13C, 15N and 1H assignments of human carbonic anhydrase II. J. Mol. Biol. **264**, 1101–16 (1996).

[24] Kay, L. E., Torchia, D. A. & Bax, A. Backbone dynamics of proteins as studied by 15N inverse detected heteronuclear NMR spectroscopy: application to staphylococcal nuclease. Biochemistry **28**, 8972–9 (1989).

[25] Palmer, A. G., Skelton, N. J., Chazin, W. J., Wright, P. E. & Rance, M. Suppression of the effects of cross-correlation between dipolar and anisotropic chemical shift relaxation mechanisms in the measurement of spin-spin relaxation rates. Mol. Phys. **75**, 699–711 (1992).

[26] Barbato, G., Ikura, M., Kay, L. E., Pastor, R. W. & Bax, A. Backbone dynamics of calmodulin studied by 15N relaxation using inverse detected two-dimensional NMR spectroscopy: the central helix is flexible. Biochemistry **31**, 5269–78 (1992).

[27] Kay, L. E., Nicholson, L. K., Delaglio, F., Bax, A. & Torchia, D. Pulse sequences for removal of the effects of cross correlation between dipolar and chemical-shift anisotropy relaxation mechanisms on the measurement of heteronuclear T1 and T2 values in proteins. J. Magn. Reson. **97**, 359–375 (1992).

[28] Grzesiek, S. & Bax, A. The importance of not saturating water in protein NMR. Application to sensitivity enhancement and NOE measurements. J. Am. Chem. Soc. **115**, 12593–12594 (1993).

[29] Peng, J. W. & Wagner, G. Mapping of spectral density functions using heteronuclear NMR relaxation measurements. J. Magn. Reson. **98**, 308–332 (1992).

[30] Dosset, P., Hus, J. C., Blackledge, M. & Marion, D. Efficient analysis of macromolecular rotational diffusion from heteronuclear relaxation data. J. Biomol. NMR **16**, 23–8 (2000).

[31] Lipari, G. & Szabo, A. Model-free approach to the interpretation of nuclear magnetic resonance relaxation in macromolecules. 1. Theory and range of validity. J. Am. Chem. Soc. **104**, 4546–4559 (1982).

[32] Vajpai, N. et al. Solution Conformations and Dynamics of ABL Kinase-Inhibitor Complexes Determined by NMR Substantiate the Different Binding Modes of Imatinib / Nilotinib and Dasatinib * . J. Biol. Chem. **283**, 18292–18302 (2008).

[33] Carper, W. R. & Keller, C. E. Direct Determination of NMR Correlation Times from SpinLattice and SpinSpin Relaxation Times. J. Phys. Chem. A **101**, 3246–3250 (1997).

[34] Bai, Y., Milne, J. S., Mayne, L. & Englander, S. W. Primary structure effects on peptide group hydrogen exchange. Proteins **17**, 75–86 (1993).

[35] Best, R. B. & Vendruscolo, M. Structural interpretation of hydrogen exchange protection factors in proteins: characterization of the native state fluctuations of CI2. Structure **14**, 97–106 (2006).

[36] Hvidt, A. & Nielsen, S. O. Hydrogen exchange in proteins. Adv. Protein Chem. **21**, 287–386 (1966).

[37] Rennella, E. et al. Equilibrium unfolding thermodynamics of beta2-microglobulin analyzed through native-state H/D exchange. Biophys. J. **96**, 169–79 (2009).
